# From diagnosis to dialogue – reconsidering the DSM as a conversation piece in mental health care: a hypothesis and theory

**DOI:** 10.3389/fpsyt.2024.1426475

**Published:** 2024-08-06

**Authors:** Lars Veldmeijer, Gijs Terlouw, Jim van Os, Sanne te Meerman, Job van ‘t Veer, Nynke Boonstra

**Affiliations:** ^1^ Department of Psychiatry, Utrecht University Medical Center, Utrecht, Netherlands; ^2^ Digital Innovation in Health, NHL Stenden University of Applied Sciences, Leeuwarden, Netherlands; ^3^ Department of Research and Innovation, KieN VIP Mental Health Care Services, Leeuwarden, Netherlands; ^4^ Department of Child and Family Welfare, University of Groningen, Groningen, Netherlands

**Keywords:** psychiatry, diagnosis, design, innovation, mental health care

## Abstract

The Diagnostic and Statistical Manual of Mental Disorders, abbreviated as the DSM, is one of mental health care’s most commonly used classification systems. While the DSM has been successful in establishing a shared language for researching and communicating about mental distress, it has its limitations as an empirical compass. In the transformation of mental health care towards a system that is centered around shared decision-making, person-centered care, and personal recovery, the DSM is problematic as it promotes the disengagement of people with mental distress and is primarily a tool developed for professionals to communicate *about* patients instead of *with* patients. However, the mental health care system is set up in such a way that we cannot do without the DSM for the time being. In this paper, we aimed to describe the position and role the DSM may have in a mental health care system that is evolving from a medical paradigm to a more self-contained profession in which there is increased accommodation of other perspectives. First, our analysis highlights the DSM’s potential as a boundary object in clinical practice, that could support a shared language between patients and professionals. Using the DSM as a conversation piece, a language accommodating diverse perspectives can be co-created. Second, we delve into why people with lived experience should be involved in co-designing spectra of distress. We propose an iterative design and test approach for designing DSM spectra of distress in co-creation with people with lived experience to prevent the development of ‘average solutions’ for ‘ordinary people’. We conclude that transforming mental health care by reconsidering the DSM as a boundary object and conversation piece between activity systems could be a step in the right direction, shifting the power balance towards shared ownership in a participation era that fosters dialogue instead of diagnosis.

## 1 Introduction

The Diagnostic Statistical Manual of Mental Disorders (DSM) has great authority in practice. The manual, released by the American Psychiatric Association (APA), provides a common language and a classification system for clinicians to communicate about people’s experiences of mental distress and for researchers to study social phenomena that include mental distress and its subsequent treatments. Before the DSM was developed, a plethora of mental health-related documents circulated in the United States (1). In response to the confusion that arose from this diversity of documents, the APA Committee on Nomenclature and Statistics standardized these into one manual, the DSM-I (2). In this first edition of the manual, released in 1952, mental distress was understood as a reaction to stress caused by psychological and interpersonal factors in the person’s life (3). Although the DSM-I had limited impact on practice (4), it did set the stage for increasingly standardized categorization of mental disorders (5).

The DSM-II was released in 1968. In this second iteration, mental disorders were understood as the patient’s attempts to control overwhelming anxiety with unconscious, intrapsychic conflicts (3). In this edition, the developers attempted to describe the symptoms of disorders and define their etiologies. They had chosen to base them predominantly on psychodynamic psychiatry but also included the biological focus of Kraepelin’s system of classification (5, 6). During the development of the DSM-III, the task force added the goal to improve the reliability — the likelihood that different professionals arrive at the same diagnosis — of psychiatric diagnosis, which now became an important feature of the design process. The developers abandoned the psychodynamic view and shifted the focus to atheoretical descriptions, aiming to specify objective criteria for diagnosing mental disorders (3). Although it was explicitly stated in DSM-III that there was no underlying assumption that the categories were validated entities (7), the categorical approach still assumed each pattern of symptoms in a category reflected an underlying pathology. The definition of ‘mental illness’ was thereby altered from what one did or was (“you react anxious/you are anxious”) to something one had (“you have anxiety”). This resulted in descriptive, criteria-based classifications that reflected a perceived need for standardization of psychiatric diagnoses (5, 6). The DSM-III was released in 1980 and had a big impact on practice (6) as it inaugurated an attempt to “re-medicalize” American psychiatry (5).

In hindsight, it is not surprising that after the release of the DSM-III, the funding for psychopharmacological research skyrocketed (8). At the same time, the debate on the relationship between etiology and description in psychiatric diagnosis continued (9). As sociologist Andrew Scull (10) showed, the election of President Reagan prompted a shift towards a focus on biology. His successor, President Bush, claimed that the 1990s were ‘the decade of the brain,’ which fueled a sharp increase in funding for research on genetics and neuroscience (10). Despite the public push for biological research, the DSM-IV aimed to arrive at a purely atheoretical description of psychiatric diagnostic criteria and was released in 1994 (11). The task force conducted multi-center field trials to relate diagnoses to clinical practice to improve reliability, which remained a goal of the design process (12). While the DSM-IV aimed to be atheoretical, researchers argued that the underlying ontologies were easily deducible from their content: psychological and social causality were eliminated and replaced implicitly with biological causality (13). In the DSM-5, validity — whether a coherent syndrome is being measured and whether it is what it is assumed to be — took center stage (10). The definition of mental disorder in the DSM-5 was thereby conceptualized as:

“… a syndrome characterized by clinically significant disturbance in an individual’s cognition, emotion regulation, or behavior that reflects a dysfunction in the psychological, biological, or developmental processes underlying mental functioning.” (14).

With the release of the DSM-5, the debate surrounding the conceptualization of mental distress started all over again, but this can be best seen as re-energizing longstanding debates around the utility and validity of APA nosology (15). Three important design goals from the DSM-III until current editions can be observed: providing an international language on mental distress, developing a reliable classification system, and creating a valid classification system.

### 1.1 The limitations of the DSM as an empirical compass

The extent to which these three design goals were attained is only partial. The development of an international language has been accomplished, as the DSM (as well as the International Classification of Diseases) is now widely employed across most Western countries. Although merely based on consensus, the DSM enables — to an extent — professionals and researchers to quantify the prevalence of certain behaviors and find one or more classifications that best suit these observed behaviors. To this date, the expectation that diagnostic criteria would be empirically validated through research has not yet been fulfilled (10, 16, 17). As stated by the authors of the fourth edition (11), the disorders listed in the DSM are “valuable heuristic constructs” that serve a purpose in research and practice. However, it was already emphasized in the DSM-IV guidebook that they do not precisely depict nature as it is, being characterized as not “well-defined entities” (18). Furthermore, while the fifth edition refers to “syndromes,” it is again described that “there is no assumption that each category of mental disorder is a completely discrete entity with absolute boundaries dividing it from other mental disorders or from no mental disorder” (14). Consequently, there are no laboratory tests or biological markers to set the boundary between ‘normal’ and ‘pathological,’ thus, it cannot confirm or reject the presumed pathologies underlying the DSM classifications, thereby rendering the validity goal of the design unattained. Therefore, the reliability of the current major DSM (i.e., DSM-5) still raises concerns (19).

By focusing conceptually on mental distress as an individual experience, the DSM task forces have neglected the role of social context, potentially restricting a comprehensive clinical understanding of mental distress (20). There is mounting evidence and increased attention, however, that the social environment, including its determinants and factors, is crucial for the onset, course, and outcome of mental distress (21–27). Moreover, exposure to factors such as early life adversity, poverty, unemployment, trauma, and minority group position is strongly associated with the onset of mental distress (28, 29). It is also established that the range of ontological perspectives — what mental distress is and how it exists — is far broader than what is typically covered in prevailing scientific and educational discussions (30). These diverse perspectives are also evident in the epistemic pluralism among theoretical models on mental health problems (31).

### 1.2 The DSM is problematic in the transformation of mental health care

In the context of contemporary transformations in mental health care, the role of the DSM as an empirical instrument becomes even more problematic. In recent years, significant shifts have been witnessed in mental health care services, with a growing focus on promoting mental well-being, preventive measures, and person-centered and rights-based approaches (32). In contrast to the 1950s definition of health in which health was seen as the absence of disease, health today is defined as “the ability to adapt and to self-manage” (33), also known as ‘positive health.’ Furthermore, the recovery movement (34), person-centered care (35), and the integration of professionals’ lived experiences (36) all contributed to a more person-centered mental health care that promotes shared-decision making as a fundamental principle in practice in which no one perspective holds the wisdom. Shared decision-making is “an approach where clinicians and patients share the best available evidence when faced with the task of making decisions, and where patients are supported to consider options, to achieve informed preferences” (37). To realize and enable a more balanced relationship between professional and patient in shared decision-making, the interplay of healthcare professionals’ and patients’ skills, the support for a patient, and a good relationship between professional and patient are important to facilitate patients’ autonomy (38). Thus, mental health care professionals in the 21st century should collaborate, embrace ideography, and maximize effects mediated by therapeutic relationships and the healing effects of ritualized care interactions (39).

The DSM and its designed classifications, as well as their use in the community, can hinder a person-centered approach in which meaning is collaboratively derived for mental health issues, where a balanced relationship is needed, and where decisions are made together. We can demonstrate this with a brief example involving the ADHD classification and its criteria, highlighting how its design tends to marginalize individuals with mental distress, reducing their behavior to objectification from the clinician’s viewpoint. The ADHD classification delineates an ideal self that highly esteems disengagement from one’s feelings and needs, irrespective of contextual factors (40). This inclination is apparent in the criteria, including criterion 1a concerning inattention: “often avoids, dislikes, or is reluctant to engage in tasks that require sustained mental effort”. This indicates that disliking something is viewed as a symptom rather than a personal preference (40). Due to a lack of attention to the person’s meaning, a behavior that may be a preference of the individual can become a symptom of a disease. Another instance can be observed in criterion 2c: “often runs about or climbs in situations where it is inappropriate.” Although such behavior might be deemed inappropriate in certain contexts, many individuals derive enjoyment from running and climbing. In this way, ‘normal’ human behavior can be pathologized because there is no room for the meaning of the individual.

A parallel disengagement is evident in the DSM’s viewpoint on individuals with mental distress (40), as the diagnostic process appears to necessitate no interaction with an individual; instead, it fosters disengagement rather than engagement. For example, according to the DSM-5, when a child is “engaged in especially interesting activities,” the clinician is warned that the ‘symptoms’ may not manifest. Although it appears most fitting to assist the child by exploring their interests, clinicians are instead encouraged to seek situations the child finds uninteresting and assess whether the child can concentrate (40). If the child cannot concentrate, a ‘diagnosis’ might be made, and intervention can be initiated. This highlights that the design of the DSM promotes professionals to locate individual disorders in a person at face value without considering contextual factors, personal preferences, or other idiosyncrasies in a person’s present or history (41). It is also apparent that the term ‘symptom’ in the DSM implies an underlying entity as its cause, obscuring that it is a subjective criterion based on human assessment and interpretation (42). These factors make it difficult for the DSM in its current form to have a place in person-centered mental health care that promotes shared decision-making.

### 1.3 The problem and hypotheses

Diagnostic manuals like the DSM function similarly to standard operating procedures: they streamline decision-making and assist professionals in making approximate diagnoses when valid and specific measures are lacking or not readily accessible (43). However, the DSM is often (mis)used as a manual providing explanations for mental distress. This hinders a personalized approach that prioritizes the patient’s needs. Furthermore, this approach does not align with the principles of shared decision-making, as the best available evidence indicates that classifications are not explanations for mental distress. Also, disengagement is promoted in the design of the DSM, which is problematic in the person-centered transformation of mental health care in which a range of perspectives and human-centered interventions are needed. This paper aims to describe the position and role the DSM may have in a mental health care system that is evolving from a medical paradigm to a more self-contained profession in which there is increased accommodation of other perspectives. For this hypothesis and theory paper, we have formulated the following hypotheses:

(1) Reconsidering the DSM as a boundary object that can be used as a conversation piece allows for other perspectives on what is known about mental distress and aligns with the requirements of person-centered mental health care needed for shared decision-making;.
(2) Embracing design approaches in redesigning the DSM to a conversation piece that uses spectra of mental distress instead of classifications will stimulate the integration of diverse perspectives and voices in reshaping mental health care.

## 2 Co-creation of a real common language

The DSM originally aimed to develop a common language, and it has achieved that to some extent, but it now primarily serves as a common language among professionals. This does not align with the person-centered transformation in mental health care, where multiple perspectives come into play (32, 44). In this section, we will address our first hypothesis: reconsidering the DSM as a boundary object that can be used as a conversation piece allows for other perspectives on what is known about mental distress and aligns with the requirements of person-centered mental health care needed for shared decision-making. First, we will examine several unintended consequences of classifications. After that, we propose considering the DSM as boundary objects to arrive at a real common language in which the perspective of people with lived experience is promoted. This perspective views the DSM as a conversation piece that can be used as a subject, the meaning of which can be attributed from various perspectives where the premise is that there is not an omniscient perspective.

### 2.1 Validation, stigma, and making up people

Classifications influence what we see or do not see, what is valorized, and what is silenced (45). DSM classifications and the process of getting them can provide validation and relief for some service users, while for others, it can be stigmatizing and distressing (46, 47). The stigma people encounter can be worse than the mental problems themselves (48). The classification of people’s behaviors is not simply a passive reflection of pre-existing characteristics but is influenced by social and cultural factors. The evolution of neurasthenia serves as a fascinating illustration of the notable ontological changes in the design of the DSM, constantly reflecting and constructing reality. Initially, neurasthenia was considered a widespread mental disorder with presumed somatic roots. Still, it was subsequently discarded from use, only to resurface several decades later as a culture-bound manifestation of individual mental distress (49). Consequently, certain mental disorders, as depicted in the DSM, may not have existed in the same way as before the classifications were designed. This has been called ‘making up people’, which entails the argument that different kinds of human beings and human acts come into being hand in hand with our invention of the categories labeling them (50). Furthermore, it is important to consider that whether behavior is deemed dysfunctional or functional is always influenced by the prevailing norms and traditions within a specific society at a given time. Therefore, the individual meaning of the patient in its context is always more important than general descriptions and criteria of functional and dysfunctional behavior (i.e., ADHD climbing example).

Individuals might perceive themselves differently and develop emotions and behaviors partly due to the classifications imposed upon them. Over time, this can result in alterations to the classification itself, a phenomenon referred to as the classificatory looping effect (51). Moreover, when alterations are made to the world that align with the system’s depiction of reality, ‘blindness’ can occur (45). To illustrate, let’s consider an altered scenario of Bowker and Star (45) in which all mental distress is categorized solely based on physiological factors. In this context, medical frameworks for observation and treatment are designed to recognize physical manifestations of distress, such as symptoms, and the available treatments are limited to physical interventions, such as psychotropic medications. Consequently, in such a design, mental distress may solely be a consequence of a chemical imbalance in the brain, making it nearly inconceivable to consider alternative conceptualizations or solutions. Thus, task forces responsible for designing mental disorder classifications should be acutely aware that they actively contribute to the co-creation of reality with the classifications they construct upon reality (49).

### 2.2 Reification and disorderism

Another unintended consequence is the reification of classifications. Reification involves turning a broad and potentially diverse range of human experiences into a fixed and well-defined category. Take, for example, the case of the classification of ADHD and its reification mechanisms (i.e., language choice, logical fallacies, genetic reductionism, and textual silence) (42). Teachers sometimes promote the classification of ADHD as they believe it acknowledges a prior feeling that something is the matter with a pupil. The classification is then seen as a plausible explanation for the emergence of specific behaviors, academic underperformance, or deviations from the expected norm within a peer group (52, 53). At first glance, this may seem harmless. However, it reinforces the notion that a complex and multifaceted set of contextual behaviors, experiences, and psychological phenomena are instead a discrete, objective entity residing in the individual. This is associated with presuppositions in the DSM that are not explicitly articulated, such as attributing a mental disorder to the individual rather than the system, resulting in healthcare that is organized around the individual instead of organized around the system (54).

In this way, DSM classifications can decontextualize mental distress, leading to ‘disorderism’. Disorderism is defined as the systemic decontextualization of mental distress by framing it in terms of individual disorders (55). The processes by which people are increasingly diagnosed and treated as having distinct treatable individual disorders, exemplified by the overdiagnosis of ADHD in children and adolescents (56), while at the same time, the services of psychiatry shape more areas of life, has been called the ‘psychiatrization of society’ (57). The psychiatrization of society encompasses a pervasive influence whereby the reification and disorderism extend beyond clinical settings and infiltrate various facets of daily life. It is a double-edged sword that fosters increased awareness of mental health issues and seeks to reduce stigma, but at the same time, raises concerns about the overemphasis on medical models, potentially neglecting the broader social, cultural, and environmental factors that contribute to individual well-being as well as population salutogenesis (58).

### 2.3 The DSM as a boundary object between activity systems in clinical practice

Instead of using the DSM as a scientific and professional tool in order to classify, the DSM can be reconsidered as a boundary object. When stakeholders with different objectives and needs have to work together constructively without making concessions, like patients and professionals in person-centered mental health care, objects can play a bridging role. Star and Griesemer (59) introduced the term boundary objects for this purpose.

“Boundary objects are objects that are plastic enough to adapt to the local needs and constraints of the different parties using them, yet robust enough to maintain a common identity in different locations. They are weakly structured in common use and become strongly structured in use in individual locations. They can be abstract or concrete. They have different meanings in different social worlds, but their structure is common enough to more than one world to make them recognizable, a means of translation.” (59).

Before exploring the benefits of a boundary object perspective for the DSM, it is important to note that it remains questionable whether the DSM in its current form can help establish a shared understanding or provide diagnostic, prognostic, or therapeutic value (60–63). To make the DSM more suitable for accommodating different perspectives and types of knowledge, the DSM task force can focus its redesign on leaving the discrete disease entities — which classifications imply — behind by creating spectra. This way of thinking has already found its way to the DSM-5, in which mental distress as a spectrum was introduced in the areas of autism, substance use, and nearly personality disorders, and following these reconceptualization, also a psychosis spectrum was proposed (43), but this proposition was eventually not adopted in the manual. As mental distress can be caused by an extensive range of factors and mechanisms that result from interactions in networks of behaviors and patterns that have complex dynamics that unfold over time (64), spectra of mental distress may be more suitable for conversations about an individual’s narrative and needs in clinical practice, as each experience of mental distress is unique and contextual.

**Figure 1:**
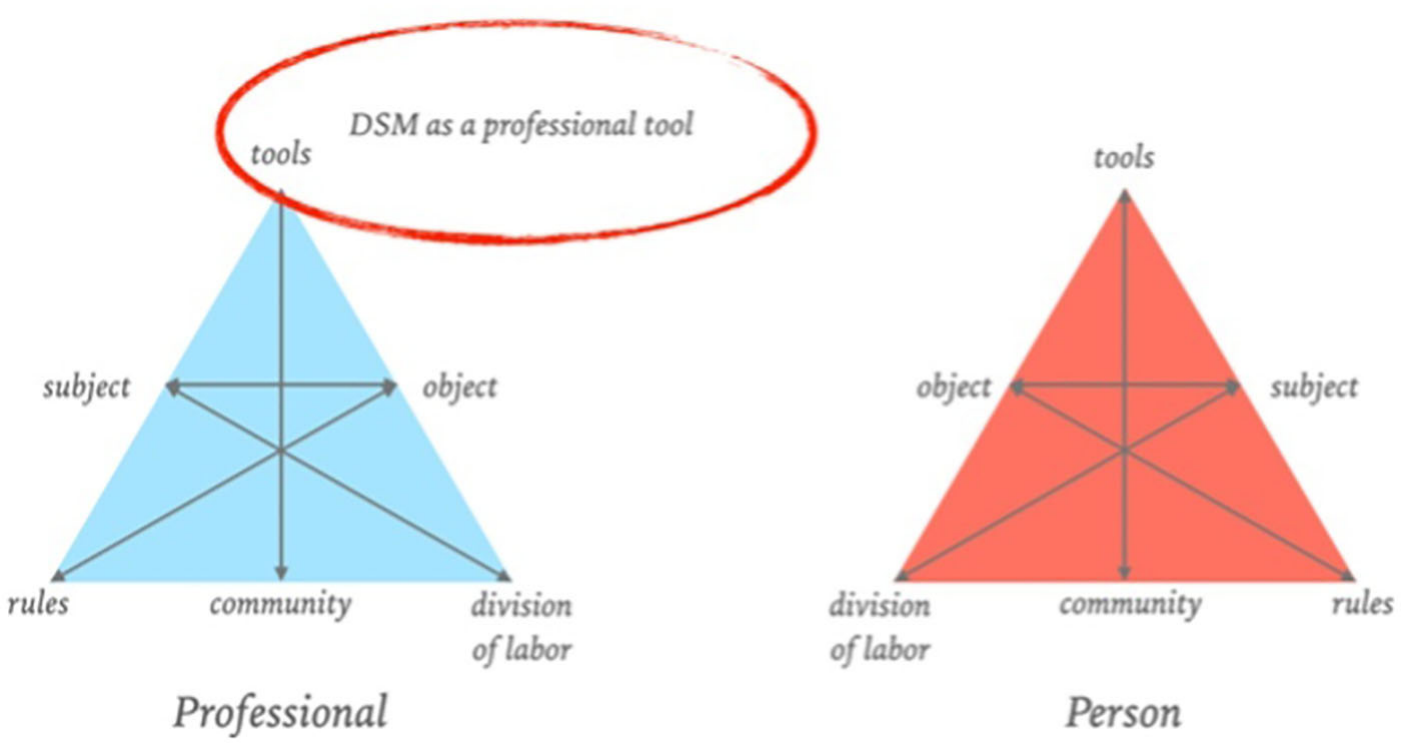
DSM as a professional tool, adapted from Figure 1, ‘Design of a Digital Comic Creator (It’s Me) to Facilitate Social Skills Training for Children With Autism Spectrum Disorder: Design Research Approach’, by Terlouw et al., CC-BY (65).

If the DSM is reconsidered as a boundary object that is intended to provide a shared language for interpreting mental distress while addressing the unintended consequences of classifications, it is also essential to consider where this language now primarily manifests itself, how it relates to shared decision-making, and the significant role it plays for patients in the treatment process. In recent decades, the DSM has positioned itself primarily as a professional tool for clinical judgment (see **Figure 1**). In this way, professionals have more or less acquired a monopoly on the language of classifications and the associated behaviors and complaints described in the DSM. It provides professionals with a tool to pursue their professional objectives and legitimacy for their professional steps with patients, resulting in a lack of equality from which different perspectives can be examined side by side. However, with shared decision-making, patients are expected to be engaged and to help determine the course of treatment; the language surrounding classifications and symptoms does not currently allow that to happen sufficiently.

**Figure 2:**
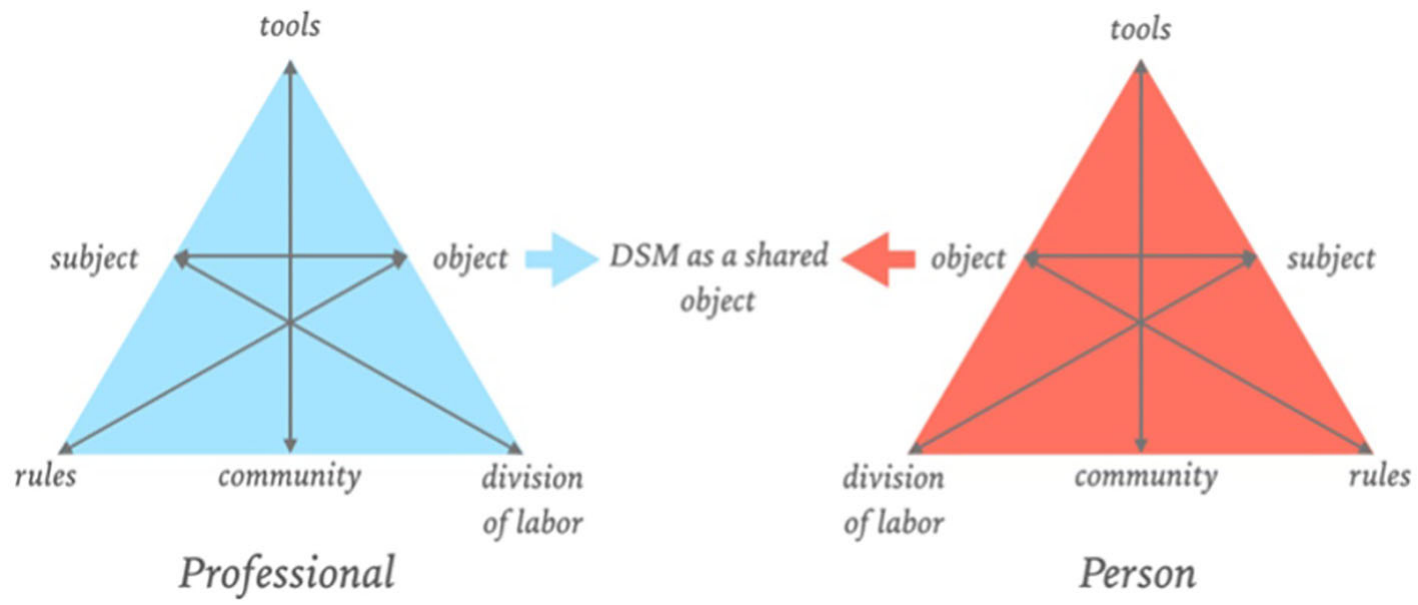
DSM as a boundary object, adapted from Figure 1, ‘Design of a Digital Comic Creator (It’s Me) to Facilitate Social Skills Training for Children With Autism Spectrum Disorder: Design Research Approach’, by Terlouw et al., CC-BY (65).

This is where boundary objects come into play. The focused shaping of boundary objects can ensure a more equal role for different stakeholders (65–67). Boundary objects can also trigger perspective-making and -taking from a reflective dialogical learning mechanism (68–70), which ensures a better shared understanding of all perspectives. Boundary objects and their dialogical learning mechanisms also align well with co-design (71). If we consider the DSM a boundary object, it positions itself between the activity system of the professionals, patients, and other people close to the patient (**Figure 2**). The boundary between activity systems represents not only the cultural differences and potential challenges in actions and interactions but also the significant value in establishing communication and collaboration (71). All sides can give meaning to the DSM language from their perspective. By effectively considering the DSM as a boundary object, the DSM serves as a conversation piece—a product that elicits and provides room for questions and comments from other people, literally one that encourages conversation (72). As a conversation piece rather than a determinative classification system, it can contribute to mapping the meaning of complaints, behaviors, signs, and patterns for different invested parties. It also provides space for the patient’s contextual factors, subjective experience, needs, and life events, which are essential to giving constructive meaning to mental distress. This allows for interpretative flexibility; professionals can structure their work, while patients can give meaning to their subjective experience of mental distress.

As the DSM as a boundary object enables interpretative flexibility, it could then be used to enact conversations and develop a shared understanding in partnership between the patient and the professional; patients are no longer ‘diagnosed’ with a disorder from a professional point of view. It is important to note that the conceptual history of understanding the diagnostic process as essentially dialogical and not as a merely technical-quantitative procedure was already started in the early 1900s. For example, in the 1913 released *‘General Psychopathology,’* Karl Jaspers presented a phenomenological and comprehensive perspective for psychiatry with suggestions about how to understand the psychopathological phenomena as experienced by the patient through empathic understanding, allowing to understand the patient’s worldview and existential meanings (73). A century after its first publication, academics continue to leverage Jaspers’ ideas to critique modern operationalist epistemology (74). Following the notion of the diagnostic process as a dialogical one, the reconsideration of the DSM as a boundary object could accommodate the patient’s idiographic experience and the professional’s knowledge about mental distress by using these potential spectra as conversation pieces, shifting the power balance in clinical practice towards co-creation and dialogue. The spectra can then be explained as umbrella terms that indicate a collection of frequently occurring patterns and signs that can function as a starting point for a co-creative inquiry that promotes dialogue, aligning more with current empirical evidence of lived experience than using classifications as diagnoses.

Considering the advantages and strengths boundary objects bring to a mental health care system centered around shared decision-making and co-creation, the DSM could be a boundary object that is interpreted from various perspectives. Take, for example, altered perceptions, which is a characteristic commonly seen in people who receive a psychosis-related classification in clinical care. For some, these perceptions have person-specific meaning (75, 76). By using the DSM as a boundary object and as a conversation piece, the patient and professional can give meaning by using the spectra in the manual as a starting point for a common language instead of using a classification to explain the distress. This requires a phenomenological and idiographic approach considering person-specific meaning and idiosyncrasies. Consequently, diagnostic practices should be iterative to align with the dynamic circumstances, with the individual’s narrative taking center stage in co-creation between professional and patient (41, 49), as this reconsidered role fosters the engagement instead of the disengagement of patients. Additionally, the potential role of the DSM as a boundary object and conversation piece may also have a positive effect on societal and scientific levels, specifically on how mental distress is perceived and conceptualized. It can ‘systemically contextualize’ mental distress, which could eliminate the disorderism and the psychiatrization of society, and in the end, hopefully, contribute to population salutogenesis.

## 3 Co-design of DSM spectra of mental distress

If the DSM is reconsidered as a conversation piece in which spectra of mental distress replace classifications, it is important to address that these must be co-designed to accommodate diverse stakeholder perspectives and various types of knowledge side by side in clinical practice. Therefore, developers and designers need to embrace lived experience in the co-development of these spectra of mental distress to ensure patients’ engagement in clinical practice, as the patient effectively becomes a stakeholder of the DSM. This requires a different approach and procedure than DSM task forces used in past iterations. In this section, we will address our second hypothesis: embracing design approaches in redesigning the DSM to a conversation piece that uses spectra of mental distress instead of classifications will stimulate the integration of diverse perspectives and voices in reshaping mental health care. While we focus a little on the what (spectra of mental distress), we mainly focus on the how (the procedure that could be followed to arrive at the what). First, we will discuss the importance of lived experience leadership in design and research. Second, we argue that in the conceptual co-design of DSM spectra, lived experience leadership can be a way forward. Third, we take the stance that a designerly way of thinking and doing can shift the premature overcommitment task forces had to iterative exploration. In the concluding paragraph, we propose a design procedure that embraces engagement and iteration as core values for developing robust and flexible spectra of mental distress that are meaningful for service users and professionals.

### 3.1 Lived experience leadership and initiatives in design and research

First, let us briefly examine the evolution of lived experience in design and science over time to provide context for why engaging people with lived experience in the design of spectra of mental distress is important for innovation. Since 1960, people with lived experiences have tried to let their voices be heard, but initially to no avail, and their civil rights movement of reformist psychiatry was labeled as ‘anti-psychiatry’ (77). During the turn of the millennium, lived experience received increased recognition and eventually became an important pillar of knowledge that informed practice and continues to do so on various levels of mental health care (34, 36, 78–81). While there is currently growing attention to the perspective of lived experience in, for example, mental health research (79, 80, 82, 83) and mental health care design and innovation (84–90), overall, their involvement remains too low in the majority of research and design projects (88, 91, 92). While there has been a significant increase in the annual publication of articles claiming to employ collaborative methods with people with lived experience, these studies often use vague terms to suggest a higher engagement level than is the case (93). This has led to initiatives such as that of The Lancet Psychiatry to facilitate transparent reporting of lived experience work (93, 94).

Although the involvement of people with lived experience and its reporting needs attention in order to prevent tokenism and co-optation (89), some great user-driven initiatives resulted in innovative design and research that improved mental health care and exemplifies why their engagement should be mandatory. The Co-Design Living Labs is such an initiative. Its program exemplifies an adaptive and embedded approach for people with lived experiences of mental distress to drive mental health research design to translation (95). In this community-based approach, people with lived experience, their caregivers, family members, and support networks collaboratively drive research with university researchers, which is very innovative considering the relatively low engagement of people with lived experience in general mental health research. Another example is the development of person-specific tapering medication initiated by people with lived experience of withdrawal symptoms. People with lived experience began to devise practical methods to discontinue medications on their own safely because of the lack of a systematic and professional response to severe and persistent withdrawal. This resulted in the accumulation of experience-based knowledge about withdrawal, ultimately leading to co-creating what is now known as tapering strips (81). The development of these tapering strips shows that people with lived experience have novel experience-based ideas for design and research that can result in human-centered innovation. Both examples underline the importance of human-centered design in which people with lived experience and knowledge are taken seriously and why the participation era requires that individuals with lived experience are decision-makers from the project’s start to produce novel perspectives for innovative design and research (88, 93).

### 3.2 The conceptual co-design of DSM spectra of mental distress and the potential of integrating lived experiences

Engaging people with lived experience of mental distress in redesigning the DSM towards a spectrum-based guideline is of special importance, albeit a more conceptual design task in comparison to the earlier examples. What mental distress is remains a fundamental philosophical and ontological question that should be addressed in partnership as it sits at the core of how mental health care is organized. To allow novel ontologies to reach their full potential and act as drivers of a landscape of promising innovative scientific and clinical approaches, investment is required in development and elaboration (30). This, as well as the epistemic pluralism among theoretical models on mental health problems (31), makes it evident there is currently not one coherent accepted explanation or consensus on what mental distress is and how it exists. Without clear etiological understanding, the most logical first step should be to involve people with lived experience of mental distress in the redevelopment of the DSM. Accounts from people with lived experience of mental distress are directly relevant to the design of the DSM, as they provide a more comprehensive and accurate understanding of mental distress and its treatment (96). Moreover, the DSM’s conceptualization as a major determinative classification system could be standing at the core of psychiatry’s “identity crisis”, where checklists of symptoms replaced thoughtful diagnoses despite after decades of brain research, no biomarker has been established for any disorders defined in the DSM (10, 97).

Design approaches can help DSM task forces prioritize integrating lived experiences to co-create a framework that can accommodate a range of perspectives to make it viable as a conversation piece. As DSM classifications do not reflect reality (98), listening to people with firsthand experiences is necessary. The CHIME framework – a conceptual framework of people’s experiences of recovery – shows, for example, a clear need to diagnose not solely based on symptoms but also considering people’s stages in their journey of personal recovery (80). Further, bottom-up research shows that the lived experience perspective of psychosis can seem very different compared to conventional psychiatric conceptualizations (82). This is also the case for the lived experience of depression (99). Design approaches can ensure that such much-needed perspectives and voices are adhered to in developing meaningful innovations (88), which brings us back to the design of the DSM. Although the DSM aims to conceptualize the reality of mental distress, engaging people with experiences of living with mental distress has never been prioritized by the DSM task force as an important epistemic resource. This is evidenced by the historically low engagement of people with lived experiences and their contexts. For example, although “individuals with mental disorders and families of individuals with mental disorders” participated in providing feedback in the DSM-5 revisions process (14), when and how they were involved, what feedback they gave, and how this was incorporated are not described. According to the Involvement Matrix (100) — a matrix that can be used to assess the contribution of patients in research and design —, giving feedback can be classified as ‘listeners’ or ‘co-thinkers,’ which are both low-involvement roles. Moreover, a review of the members of the DSM task forces and working groups listed in the introductions of the DSMs shows patients have never been part of the DSM task force and thus never been part of the decision-making process (96). Human-centered design is difficult to achieve when people with lived experience are not involved from preparation to implementation but are only asked to give feedback on expert consensus (88).

In the participation era, using a design approach in mental health care without engaging important stakeholders can be problematic. For example, it is evident that the involvement of people with lived experience changes the nature of an intervention dramatically, as people’s unique first-hand experiences, insights about mental states, and individual meaning and needs are often different in design activities as opposed to what general scientific and web-related resources suggest (101, 102). Further, clear differences are reported around designers, researchers, and clinicians on one side and service user ideas of meaningful interventions on the other (102, 103). Thus, the meaningful engagement of people with lived experience in design processes always exposes gaps between general research and the interests and lives of service users (104). This makes the participation of people with lived experience in developing innovative concepts — and, as such, in the conceptual design of DSM spectra of mental distress — essential because their absence in design processes may lead to ineffective outcomes (102). This design perspective may explain some of the negative effects of the DSM. The classifications aimed to be empirical constructs reflecting reality, yet phenomena such as reification and the classificatory looping effect emerged (42, 51). From a design perspective, the emergence of these effects may have a simpler explanation than previously presumed: the premature over-commitment in the DSM’s design processes without input from individuals with firsthand experiences.

### 3.3 Shifting the premature over-commitment to iterative exploration

The centrist development approach used to design the DSM implicitly frames people with mental distress as ‘ordinary people,’ resulting in ‘average solutions’ because their experiences are decontextualized and lumped together on a group level — eventually leading to general descriptions for a universal appliance. Instead, a more human-centered iterative design process in which people with lived experience play an important role, preferably as decision-makers, can promote the design of spectra of mental distress that leave room for idiosyncrasies that correspond with people’s living environments on an individual level. This can potentially ensure that they are actually helpful for shared decision-making between patients and professionals and resonate in person-centered mental health care. A design approach is feasible for this aim because design processes are not searching for a singular ‘truth’ but rather exploring the multiple ‘truths’ that may be relevant in different contexts (105). This can be of added value to conceptualizing spectra of mental distress, which is known to have characteristics that overlap between people but also to have a unique phenomenology and contextual foundation for each individual — in the case of mental distress, there literally are multiple truths dependable on who and what you ask in what time and place. Furthermore, design approaches enable exploration and discovery (106). Designers consistently draw cues from the environment and introduce new variables into the same environment to eventually discover what does and does not work (107). This explorative attitude also ensures the discovery of unique insights, such as people’s experiential knowledge and contexts. Therefore, from a design perspective, predetermining solutions might be ineffective for arriving at DSM innovation. This is, for example, aptly described by Owens et al. (101):

“… the iterative nature of the participatory process meant that, although a preliminary programme for the whole workshop series was drawn up at the outset, plans had to be revised in response to the findings from each session. The whole process required flexibility, a constantly open mind and a willingness to embrace the unexpected”.

These insights illustrate the core of design that can guide the development of future DSM iterations: design enables the task force to learn about mental health problems without an omniscient perspective by iteratively developing and testing conceptualizations in the environment in partnership with the target group. As participatory design studies consistently demonstrate, solutions cannot be predetermined solely based on research and resources. The involvement of individuals with lived experience and their contexts invariably uncovers crucial serendipitous insights that challenge the perspectives on the problem. This can expose important misconceptions, such as the tendency to underestimate the complexity of human experience and decontextualize it from its environment.

### 3.4 Insights that could inform a procedure for co-designing spectra of mental distress

People with lived experience need to be highly involved in developing meaningful spectra of mental distress to guide conversations in clinical practice. As we now have a comprehensive understanding of what design approaches can offer to the development procedure of a lived experience-informed DSM, we will highlight these insights in this paragraph.

#### 3.4.1 Balance academic research with lived experience insights

In the design procedure of a future DSM, academic research can be used to learn about people’s experiences of mental distress but never as the source alone for the development of spectra of mental distress. In this way, designers and researchers in mental health care need to involve people with lived experience at the heart of design processes as partners and come to unique insights together without an omniscient perspective. The aim should not be to design general descriptions but to design spectra that are flexible enough to adapt to local needs and constraints for the various parties using them yet robust enough to maintain a common identity across different locations. This allows the DSM to have different meanings in different social worlds, while at the same time, their structure is common enough for more than one world to recognize them.

#### 3.4.2 Prevent premature overcommitment in the design process

Conceptualizations of spectra of mental distress must not be predetermined, and there should be no overcommitment to concepts in the early phases of the project. Thus, the task force should avoid viewing mental distress too narrowly, too early on in the process. This enables the evolution of lived experience-based spectra in an iterative design- and test process. The starting point should be an open representation of mental distress and discover together with people with lived experience how this could be best conceptualized and what language should be used. This allows room for exploring and discovering what works and aligns with patients’ needs and experiences in their living environments and professionals’ needs in their work environments.

#### 3.4.3 Designing and testing is also a form of research

Researchers and designers should realize that designing and testing conceptualizations in partnership with people with lived experience also results in unique knowledge that can guide the development — designing and testing the developed concepts is a form of research. For example, exploring if a certain designed spectrum resonates as a conversation piece between patients and professionals in clinical practice provides qualitative insights that cannot be predicted beforehand. In this way, science and design can complement the innovation of the DSM: science benefits from a design approach, while design benefits from scientific methods (108). Flexible navigation between design and science would indicate that the developed DSM can be meaningful as a conversation piece in clinical practice.

#### 3.4.4 Good design comes before effective science

Good design comes before effective science, as innovations are useless if not used, even if they are validated by science (85). Although the development of the DSM is often described as a scientific process, our analysis indicates that it is more accurately described as a design process. As a design process, it requires a methodologically sound design approach that is suitable for involving patients and people with lived experience. Co-design is a great contender for this purpose, as a systematic review showed this approach had the highest level of participant involvement in mental health care innovation (89). Although people with lived experience have never been involved as decision-makers, this should be the aim of the design process of a novel DSM in the participation era. This promotes lived experience leadership in design and, ultimately, contributes to more effective science.

#### 3.4.5 Avoid tokenism and co-optation

Involving people with lived experience as decision-makers in redesigning the DSM must avoid tokenism and co-optation and address power imbalances. The first step that the task force can take is to use the Involvement Matrix (100) together with people with lived experiences to systematically and transparently plan, reflect, and report on everyone’s contribution to the design process. This has not been prioritized in the past DSM revisions. In the end, transparency and honesty about collaboration can support the empowerment of people with firsthand perspectives and shift the power imbalance towards co-creation for more human-centered mental health care. This is needed, as the involvement of people with lived experience in design and research processes is currently too low and obscured by vague terms and bad reporting.

## 4 Discussion and conclusion

In this hypothesis and theory paper, we have argued that the current role of the DSM, as an operating manual for professionals, can be reconsidered as a boundary object and conversation piece for patients and professionals in clinical practice that stimulates dialogue about mental distress. In this discussion, we will address five themes. First, while we argued that research acknowledges the absence of empirical support for biological causation, we believe characterizing the DSM as entirely non-empirical may be incorrect. Second, we discuss our perspective on balancing between a too-narrow medical perspective and a too-broadly individualized perspective. Third, we discuss why mental health care also needs novel methods for inquiry if the DSM is reconsidered as a conversation piece. Fourth, we discuss that while we are certain that design approaches can be fruitful for redesigning the DSM, some challenges regarding tokenism, co-optation must be addressed. We conclude by examining various methodological challenges and offering recommendations for the co-design process of the DSM.

### 4.1 Redesigning instead of discarding the DSM

The DSM is too deeply entrenched in mental health care to discard it simply. The DSM is embedded in not only mental health care but also society. For instance, a DSM classification is necessary in the Netherlands to get mental health care reimbursement, qualify for additional education test time, or receive subsidized assisted living. Moreover, it is ingrained in research and healthcare funding, making it unproductive and somewhat dangerous to discard without an alternative, as it may jeopardize access to care and impact insurance coverage for treatment and services that people with mental distress need. Therefore, we posited that instead of discarding the DSM, its role should be reconsidered in a mental health care system centered around shared decision-making and co-creation to eliminate pervasive effects such as the disengagement of patients, reification, disorderism, and the psychiatrization of society. However, the DSM categories are not entirely *a priori* constructed as is sometimes claimed, as the psychiatric symptom space and diagnostic categories took shape in the late nineteenth century through decades of observation (109).

While this adds important nuance to the idea that the design of the DSM is entirely non-empirical, it does not invalidate the argument that the DSM design is grounded in a potentially false ontology (64). Though the lack of evidence does not necessarily indicate evidence of absence, and the biological context in some way plays a role, research shows various other dimensions of life — including the social, historical, relational, environmental, and more — also influence mental distress, yet are significantly underemphasized in its current design. We believe that we showed this manifests itself most prominently in the various highly arbitrary classification designs that can confuse the professional and the patient and appear limited in providing meaningful guidance for clinical practice, design, and research. That is why we have proposed redesigning the next iteration of the DSM to primarily focus on formulating a set of spectra of distress. Reconsidering the DSM leverages one of its biggest strengths: the DSM is not bound by an analytic procedure but rather is guided by scientific debate (17). Further, developments and amendments to psychiatric classification systems have always reflected wider social and cultural developments (110). The recognition, implementation, and impact of the DSM in Western countries can even be seen as a reason not to focus on developing alternative models but rather to redesign the DSM so that it conceptually aligns with the social developments, scientific findings, and needs of people in the 21st century, as it is already deeply embedded in systems. Given that DSM classifications are now recognized as inaccurate depictions of the reality of mental distress (98) and that, at the same time, mental health care is shifting towards person-centeredness and shared decision-making, we believe the proposals in this article are not radical but rather the most meaningful way forward to accommodate diverse perspectives.

### 4.2 Balancing between a too-narrow medical categorization and a too-broadly individualized approach

From a classical psychopathological perspective, integrating the lived experiences of those with mental distress into the redevelopment of the DSM as a boundary object presents certain conceptual challenges. For example, uncritically overemphasizing individual experiences might lead to an underappreciation of psychopathological manifestations like, for example, altered perceptions. Conversely, excluding people with lived experience from the DSM’s design processes has resulted in its own conceptual and epistemic issues, such as undervaluing the idiographic, contextual, and phenomenological aspects of individual mental distress. Therefore, we argue that achieving a balance between these differing but crucial perspectives should result from a co-design procedure for a revised DSM. Determining this balance before obtaining results from such a process is too premature and arbitrary and would contradict our recommendation to prevent over-commitment in the early stages of the design process. As people with lived experience were never previously involved, it is impossible to predict the outcomes of a co-design procedure or hypothesize about a clear distinction between these perspectives in the DSM’s conceptual development beforehand. As seen in past iterations, prematurely drawing rigid lines could hinder the design process and result in design fixation. From the perspective of boundary objects, the DSM cannot have one dominant perspective if it is to function effectively. All stakeholders must be able to give meaning to the spectra of mental distress from their own activity systems, and these perspectives should be equal in order to create a shared awareness of the different perspectives involved. A DSM designed as a boundary object triggers dialogical learning mechanisms, ensuring the multiple perspectives are harmonized rather than adjusted to fit one another, ensuring no single perspective prevails over the others or consensus is pursued (71, 111).

### 4.3 Novel methods for inquiry to accompany the reconsidered role of the DSM

If the DSM is reconsidered and designed as a conversation piece and classifications are replaced by spectra, in clinical practice, a unique language needs to be co-developed between the patient and the professional, and an equal relationship is important to ally. For example, if we consider the person-specific meanings of altered perceptions, they need to be explored, as they have clinical relevance. However, for such purposes, current diagnostic methods in clinical practice are limiting because they are highly linguistic and tailored to classification systems and the needs and praxis of the professionals. This can impede the DSM’s effectiveness as a tool for dialogue. Expressing the uniqueness of an experience of mental distress is difficult — especially during a mental crisis — let alone effectively communicating it to a professional. While people with mental distress can effectively communicate their behaviors and complaints, which fits the current use of the DSM, people have far more embodied and experiential knowledge of their distress. How people cope with their mental distress in the contexts they are living in is very difficult to put into words without first making these personal and contextual insights tangible (41), yet this is essential information for when the DSM is used as a boundary object and conversation piece. To accommodate the patient in making this knowledge tangible, the professional becomes more of a facilitator than an expert, emphasizing therapeutic relationships and the healing effects of ritualized care interactions (39). This transformation requires novel co-creative methods for inquiry (41) and professional training (39). Therefore, expanding the diagnostic toolkit with innovative and creative tools and embracing professionals such as art therapists, social workers, and advanced nurse practitioners to enable and support patients to convey their narratives and needs in their own way is essential if the DSM is to be used as a boundary object and conversation piece.

### 4.4 Promoting lived experience leadership in the co-design procedure

Despite longstanding calls for the APA to include people with lived experience in the decision-making processes for diagnostic criteria, the DSM-5 task force did not accept this inclusion. The task force believed incorporating these perspectives could compromise objectivity in the scientific process (96). This mindset ensures that research, design, and practice remain predominantly shaped by academics and professionals, causing conventional mental health care to perpetuate itself. It continues to repeat the same approaches and consequently achieves the same results. Therefore, people with lived experience should have more influence in the participation era to accelerate change in mental health care. This proposition comes with some challenges regarding power imbalances that need addressing. While it is acknowledged that the involvement of individuals with lived experience yields unique insights and can serve as strong collaborators and knowledgeable contributors, they are never given decision-making authority in design processes in mental health care (88, 89, 92) or in the DSM’s development processes (96). This lack of authority impedes lived experience leadership (91, 112) and subsequently stands in the way of effectively reconsidering and redesigning the DSM. To avoid tokenism, the DSM revision process should not settle for low engagement and involvement but set the bar higher by redressing power imbalances (113). Furthermore, in the co-design process of the DSM, the task force should not view objectivity as the opposite of subjectivity or strive for consensus. Instead, they should value group discussions and disagreements, encouraging stakeholders to debate and explore the sources of their differing perspectives and knowledge (96). Shifting towards lived experience leadership starts with perceiving and engaging people with lived experiences of mental distress as experts of their experiences in iterative design and research processes and giving them this role in revising the DSM.

### 4.5 Methodological considerations for a co-design procedure of the DSM

Merely positioning people with lived experience as partners and decision-makers is insufficient; there are also significant methodological concerns regarding the execution of design research in mental health care. Although iteration and participation are essential for design in mental health care, as designers focus on the unmet needs of service users and ways to improve care (114), research shows design is not always executed iteratively, and end users are not always involved. For example, about one-third of projects that designed mental health interventions did not adopt an iterative process (85). The engagement of end users in design processes in mental health is also not yet a common practice. For instance, a systematic review of serious games in mental health for anxiety and depression found that only half of these games, even while reporting using a participatory approach, were designed with input from the intended end-users (115). A systematic review of design processes that aimed to design innovations for people with psychotic symptoms overlaps these findings, as less than half of the studies demonstrated a high level of participant involvement in their design processes (89).

The low level of involvement and lack of iterative approaches in mental health care design offer valuable insights for future processes. If the DSM task force aims to adopt a co-design approach, it should incorporate these lessons to enhance design effectiveness. First, the task force must understand that design has a different aim, culture, and methods than the sciences (116). The scientific approach typically implies investigating the natural world through controlled experiments, classifications, and analysis, emphasizing objectivity, rationality, neutrality, and a commitment to truth. In contrast, a design approach focuses on studying the artificial world, employing methods such as modeling, pattern formation, and synthesis, guided by core values of practicality, ingenuity, empathy, and concern for appropriateness. Second, the task force should consider the known challenges they will encounter and need to navigate to let the paradigms be complementary in practice (117). Further, the task force should consider that the nature of design is exploratory, iterative, uncertain, and a social form of inquiry and synthesis that is never perfect and never quite finished (84). This requires tolerating ambiguity and having trust (101). Lastly, more transparency in the participatory work of the task force is called for, beginning with being honest, being detailed, addressing power imbalances, being participatory in reporting the participatory approach, and being excited and enthusiastic about going beyond tokenistic engagement (118).

Despite these challenges, transforming psychiatric diagnoses by reconsidering and redesigning the DSM as a boundary object and conversation piece could be a step in the right direction. This would shift the power balance towards shared ownership in a participation era that fosters dialogue instead of diagnosis. We hope this hypothesis and theory paper can give decisive impulses to the much-needed debate on and development of psychiatric diagnoses and, in the end, contribute to lived experience-informed psychiatric epistemology. Furthermore, as a product of an equal co-production process between various disciplines and types of knowledge, this paper shows it is possible to harmonize perspectives on a controversial topic such as the DSM.

## Data availability statement

The original contributions presented in the study are included in the article/supplementary material. Further inquiries can be directed to the corresponding author/s.

## Author contributions

LV: Conceptualization, Methodology, Project administration, Writing – original draft, Writing – review & editing. GT: Conceptualization, Methodology, Visualization, Writing – original draft, Writing – review & editing. JVO: Conceptualization, Writing – original draft, Writing – review & editing. SM: Conceptualization, Writing – original draft, Writing – review & editing. JV: Writing – original draft, Writing – review & editing. NB: Writing – original draft, Writing – review & editing.
